# Ability of AI detection tools and humans to accurately identify different forms of AI-generated written content

**DOI:** 10.1186/s41077-025-00396-6

**Published:** 2025-11-22

**Authors:** Adam Cheng, Yiqun Lin, Gabriel Reedy, Christine Joseph, Samantha Wirkowski, Viviane Mallette, Vikhashni Nagesh, David Krieser, Aaron Calhoun

**Affiliations:** 1 Departments of Pediatrics and Emergency Medicine, Cumming School of Medicine, KidSIM Simulation Program, Alberta Children’s Hospital, University of Calgary, 28 Oki Drive NW, Calgary, AB T3B 6A8 Canada; 2 KidSIM Simulation Program, Alberta Children’s Hospital, Calgary, Canada; 3 Clinical Education, Faculty of Life Sciences and Medicine, King’s College London, London, England; 4 Division of Pediatric Critical Care Medicine, Norton Children’s Hospital, University of Louisville School of Medicine, Louisville, USA; 5 Fellow of Pediatric Critical Care Medicine, University of Louisville School of Medicine, Louisville, KY USA; 6 Department of Pediatrics, Section of Neonatology, Centre Hospitalier Universitaire Sherbrooke, Sherbrooke, Canada; 7 Department of Pediatrics, Cumming School of Medicine, University of Calgary, Calgary, Canada; 8 Faculty of Medicine, Dentistry and Health Sciences, University of Melbourne, Parkville, Australia; 9 Pediatric Critical Care, University of Louisville School of Medicine, Louisville, KY USA

**Keywords:** Artificial intelligence, Detection, Large language models, ChatGPT, Academic writing, Ethics

## Abstract

**Background:**

The increasing use of artificial intelligence (AI) by scholars presents a pressing challenge to healthcare publishing. While legitimate use can potentially accelerate scholarship, unethical approaches also exist, leading to factually inaccurate and biased text that may degrade scholarship. Numerous online AI detection tools exist that provide a percentage score of AI use. These can assist authors and editors in navigating this landscape. In this study, we compared the scores from three AI detection tools (ZeroGPT, PhraslyAI, and Grammarly AI Detector) across five plausible conditions of AI use and evaluated them against human assessments.

**Methods:**

Thirty open access articles published in the journals *Advances in Simulation* and *Simulation in Healthcare* prior to 2022 were selected, and the article introductions were extracted. Five experimental conditions were examined, including: (1) 100% human written; (2) human written, light AI editing; (3) human written, heavy AI editing; (4) AI written text from human content; and (5) 100% AI written from article title. The resulting materials were assessed by three open-access AI detection tools and five blinded human raters. Results were summarized descriptively and compared using repeated measures analysis of variance (ANOVA), intraclass correlation coefficients (ICC), and Bland–Altman plots.

**Results:**

The three AI detection tools were able to differentiate between the five test conditions (*p* < 0.001 for all), but varied significantly in absolute score, with ICC ranging from 0.57 to 0.95, raising concerns regarding overall reliability of these tools. Human scoring was far less consistent, with an overall accuracy of 19%, indistinguishable from chance.

**Conclusion:**

While existing AI detection tools can meaningfully distinguish plausible AI use conditions, reliability across these tools is variable. Human scoring accuracy is uniformly low. Use of AI detection tools by scholars and journal editors may assist in determining potentially unethical use but they should not be relied upon alone at this time.

**Supplementary Information:**

The online version contains supplementary material available at 10.1186/s41077-025-00396-6.

## Background

The widespread uptake of generative artificial intelligence (AI)-based large language models (LLMs) such as the Chat Generative Pre-trained Transformer (ChatGPT) platform, has transformed the way scholars create content for academic manuscripts [1–4]. Significant concerns regarding the use of AI have been raised, including hallucinations (i.e. factually incorrect responses), bias within responses, and plagiarism. Scholars are actively debating the ethical implications of utilizing AI to support the manuscript writing process [5, 6]. Despite these concerns, some scholars are currently choosing to use LLMs as manuscript writing assistants in several ways, including the generation of written content de-novo, brainstorming and idea generation, and editorial/grammatical assessment of the text. This potentially results in a spectrum of text within a single manuscript with a varying degree of AI content [3, 6, 7]. It is critically important for simulation journal editors and reviewers to differentiate between human and AI-written text as not all uses have similar ethical implications. A recently published article examines these uses and provides guidance regarding these different use cases [6]. In general, uses of AI that involve the creation of new text or ideas are more suspect from an ethical perspective.

AI detection tools are designed to detect AI-written text and differentiate it from human written text. A variety of AI detection tools are available, with studies reporting varying degrees of accuracy in detecting AI-written text [8–12]. Little is known, however, about the ability of different AI detection tools to accurately differentiate between AI-written, human written, or variations of AI-edited text within healthcare simulation scholarship. Furthermore, it is unknown if humans can accurately differentiate between different forms of AI versus human written text. A better understanding of these issues will provide needed clarity for simulation scholars, reviewers, and journal editors amidst the evolving landscape of AI and academic writing.

In this paper, we aim to describe the ability of three open-access AI detection tools to detect ChatGPT4o generated text under various usage conditions reflective of how authors are likely to employ these tools and consonant with recently published ethical models [6]. We also aimed to assess the agreement between the three AI detection tools, and to describe the accuracy and error patterns of human detection across different conditions, including analysis of false positives and negatives.

## Methods

### Article selection

Two members of our research team (A. C. and A.W.C.) selected open access articles published between 2000 to 2022 from two healthcare simulation journals: *Simulation in Healthcar*e and *Advances in Simulation*. To standardize the length of the written content, articles with introduction sections ranging between 500–600 words in length were included in the study. The publication dates of these articles preceded the launch of ChatGPT and other widely available large language models (LLMs), thus ensuring that article content was almost certainly human in origin. Fifteen articles were selected from each journal: six conceptual or innovation articles, six original research articles, and three review articles in order to provide a representative sample of thirty healthcare simulation articles for this study.

### ChatGPT4o testing conditions

We created five different testing conditions to mimic potential uses of ChatGPT or other LLMs in the academic writing process. The following conditions were used to generate introductory text for each article:

1. 100% Human Written – the introductory paragraph of the original article was taken to represent 100% human written content and utilized verbatim.
  For the remaining 4 conditions, ChatGPT4o was accessed between March 28 – April 28, 2025 to generate text using the prompts described below. A new chat was used for each condition, temperature (i.e. determines creativity and predictability of responses) was set to default, and response length was guided by prompt wording with no use of max token limits (i.e. no limit on length of input or output).
2. Human Written text, lightly edited by AI—we asked ChatGPT4o to generate an introductory paragraph for the article by lightly editing the original, human-written introduction in the paper. The following ChatGPT4o prompt was used: “Please lightly edit the following introductory paragraph for an academic journal article by checking spelling and punctuation: [insert original introduction here]”.
3. Human Written text, heavily edited by AI – we asked ChatGPT4o to generate an introductory paragraph for the article by heavily editing the original, human-written introduction in the paper. The following ChatGPT4o prompt was used: “Please edit the following introductory paragraph for an academic journal article for spelling, grammar, punctuation, sentence structure, and overall readability of the text: [insert original introduction here]”.
4. AI Written text from human content – this condition was intended to mimic the use of ChatGPT4o to write text based upon ideas and content provided by authors. The text of the original introduction of each paper was first deconstructed into bullet points by two members of our research team (A. C, A. W. C). The main ideas and content pieces within each paragraph were captured and summarized in 3 or 4 bullet points per paragraph. We then asked ChatGPT4o to generate an introductory section for the article using the bullet pointed version of the introduction. The following ChatGPT4o prompt was used: “Please write a 500 word introductory paragraph for an academic journal article entitled [insert article title], by incorporating the following content: [insert bullet pointed content]”.
5. 100% AI Written – we asked ChatGPT4o to generate an introductory paragraph for the article based upon the title of the manuscript. The following ChatGPT4o prompt was used: “Please write a 500 word introductory paragraph for an academic journal article entitled: [insert article title]”.

### Primary outcome—AI detection software performance

We used the following open access AI detection tools in this study: (1) ZeroGPT (www.zerogpt.com); (2) Phrasly AI (www.phrasly.ai); and (3) Grammarly AI Detector (www.grammarly.com/ai-detector). We selected these based on the Google search string: “What are the best AI detection tools for academic writing?”. We excluded AI detection tools that were not open access under the assumption that academic authors would be more likely to use AI detection tools that did not require a fee. Amongst four of the free AI detection tools identified in the google search, we conducted pilot testing to assess the performance of these tools. Pilot testing consisted of assessing five sample introduction sections representing 100% human written content and 100% ChatGPT4o written content, derived from five open access healthcare simulation articles published by members of our research team. These articles were not included in the larger study sample of 30 articles. One AI detection tool (www.gptzero.me) was excluded as it was unable to reliably detect 100% human written content, often categorizing human written content as primarily AI written. The remaining three open access AI detection tools (i.e. ZeroGPT, PhraslyAI, and Grammarly AI Detector) were able to reliably differentiate 100% human versus 100% AI written content and were selected for use in our study. Introductory text from the five testing conditions were then assessed by these tools to determine the percentage of text generated by AI, represented as a score from 0 to 100.

### Secondary outcome—human detection

Five blinded raters (YL, DK, VM, VN, GR) were randomly assigned the introduction section from one of the five testing conditions for each of the 30 articles. Raters are all active healthcare simulation educators and researchers, with a wide range of clinical (1–25 years of experience), research (1–25 years), and simulation expertise (1–20 years). For each introduction section, they were asked if they thought the content was: (a) 100% Human generated text; (b) Human generated text, lightly edited by AI; (c) Human generated text, heavily edited by AI; (d) AI generated text based on human ideas; and (e) 100% AI generated. If they selected (b), (c) or (d), they were then asked to estimate the percentage of text written by AI.

## Outcomes and statistical analysis

We described the AI detections scores across the five conditions using mean and 95% confidence interval (CI) for all three tools. Repeated measures analyses of variance (ANOVA) were used to evaluate differences in AI detection scores across five conditions, as rated by all three tools. Effect sizes were presented as partial $\eta^2$. Agreement between the three tools was assessed using the intraclass correlation coefficient (ICC) and Bland–Altman plots.

We also described the accuracy of human detection across all five conditions, as well as the overall accuracy. Differences in accuracy among the five conditions were evaluated using multilevel logistic regression. Bland–Altman plots and ICC were used to assess agreement between human detection and AI tool scores.

Finally, we explored the false positive (FP) and false negative (FN) rates of human detection in conditions 1 (fully human) and 2 (fully AI). A FP was defined as human written text incorrectly classified as AI generated text, and a FN was defined as AI generated text incorrectly classified as human written text. For base case analysis, we excluded all “mixed” ratings (i.e. rated as b, c, or d) from analysis. In the sensitivity analysis, we explored 2 extreme scenarios: one in which all “mixed” ratings were considered correct (best-case scenario), and another in which they were considered error (worst-case scenario).

## Results

### Performance of AI detection tools

**Fig. 1:**
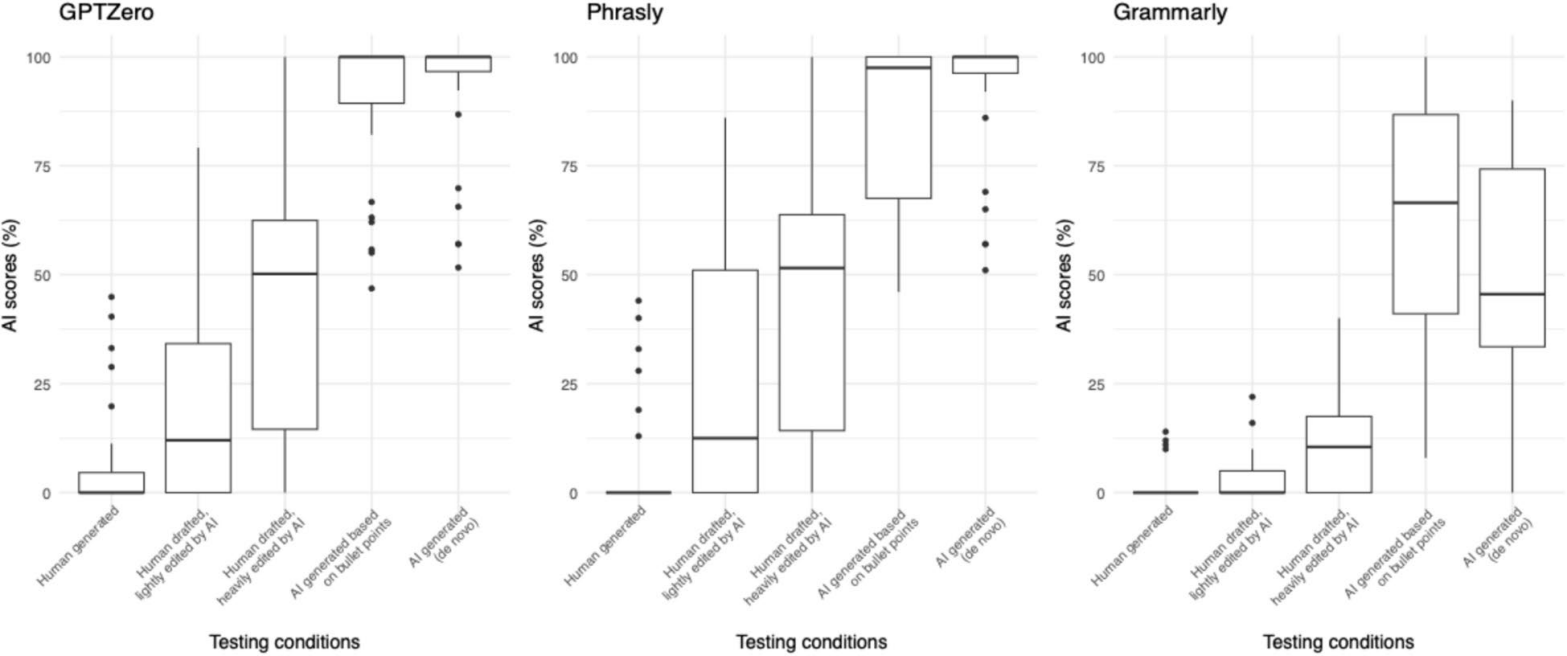
AI detection scores across 5 conditions

All tools were able to detect an increasing percentage of AI content with the exception of Grammarly’s analysis of Condition 5, which identified a wholly AI written text as only 50% AI generated. Interestingly, some AI content was detected in the fully human condition (1.6–6.5% AI content detected on average) and some human content was detected in the fully AI condition (50–92.5% AI content detected on average). Repeated measures ANOVA showed statistically significant differences among the five conditions for AI detection scores generated by ZeroGPT (partial $\eta^2$ = 0.84, *p* < 0.001), Phrasly (partial $\eta^2$ = 0.79, *p* < 0.001), and Grammarly (partial $\eta^2$ = 0.75, *p* < 0.001) (Fig. 1). Mean scores and their 95% confidence intervals (CIs) for each of the five conditions are presented in Table 1.

**Table 1:** AI detection scores across 5 conditions

		mean (95%CI)	
Condition	ZeroGPT	Phrasly	Grammarly
Human generated text	6.5 (1.6, 11.3)	5.9 (1.0, 10.8)	1.6 (0.0, 3.1)
AI lightly edited	20.2 (11.0, 29.3)	24.8 (13.9, 35.7)	3.0 (0.9, 5.1)
AI heavily edited	43.1 (32.0, 54.2)	45.1 (33.5, 56.6)	11.5 (7.2, 15.8)
AI generated based on bullet points	89.9 (83.6, 96.2)	85.6 (78.6, 92.6)	62.5 (52.5, 72.6)
AI generated (de novo)	92.5 (86.8, 98.2)	92.4 (86.7, 98.1)	50.0 (40.5, 59.5)
Effect size (partial $\eta^2$)	0.84	0.79	0.75
*p*-value	< 0.001	< 0.001	< 0.001

The agreement between ZeroGPT and Phrasly AI detection scores was very good, with an ICC of 0.96. The Bland–Altman plot indicated a bias of –0.32 and limits of agreement ranging from –22.5 to + 21.9. In contrast, the agreement between ZeroGPT and Grammarly was moderate, with an ICC of 0.60. The Bland–Altman plot showed a bias of 24.7, suggesting that ZeroGPT tends to assign higher AI detection scores than Grammarly. The 95% limits of agreement ranged from –25.4 to + 74.8, indicating proportional bias. A similar pattern was observed between Phrasly and Grammarly (ICC = 0.57, bias = 25.0, limits of agreement from –28.7 to + 78.7) (eFig. 1, Additional File).

### Human detection

**Fig. 2:**
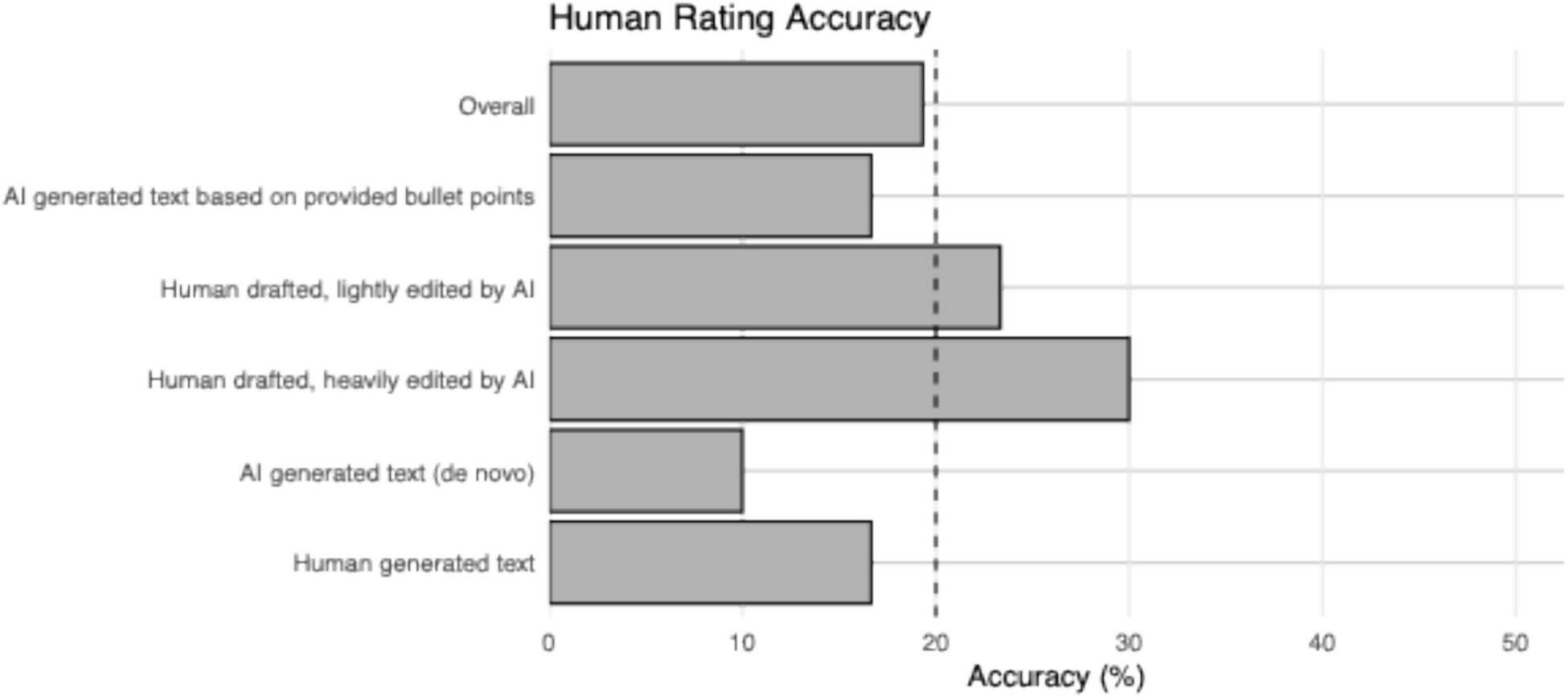
Accuracy of human detection

**Fig. 3:**
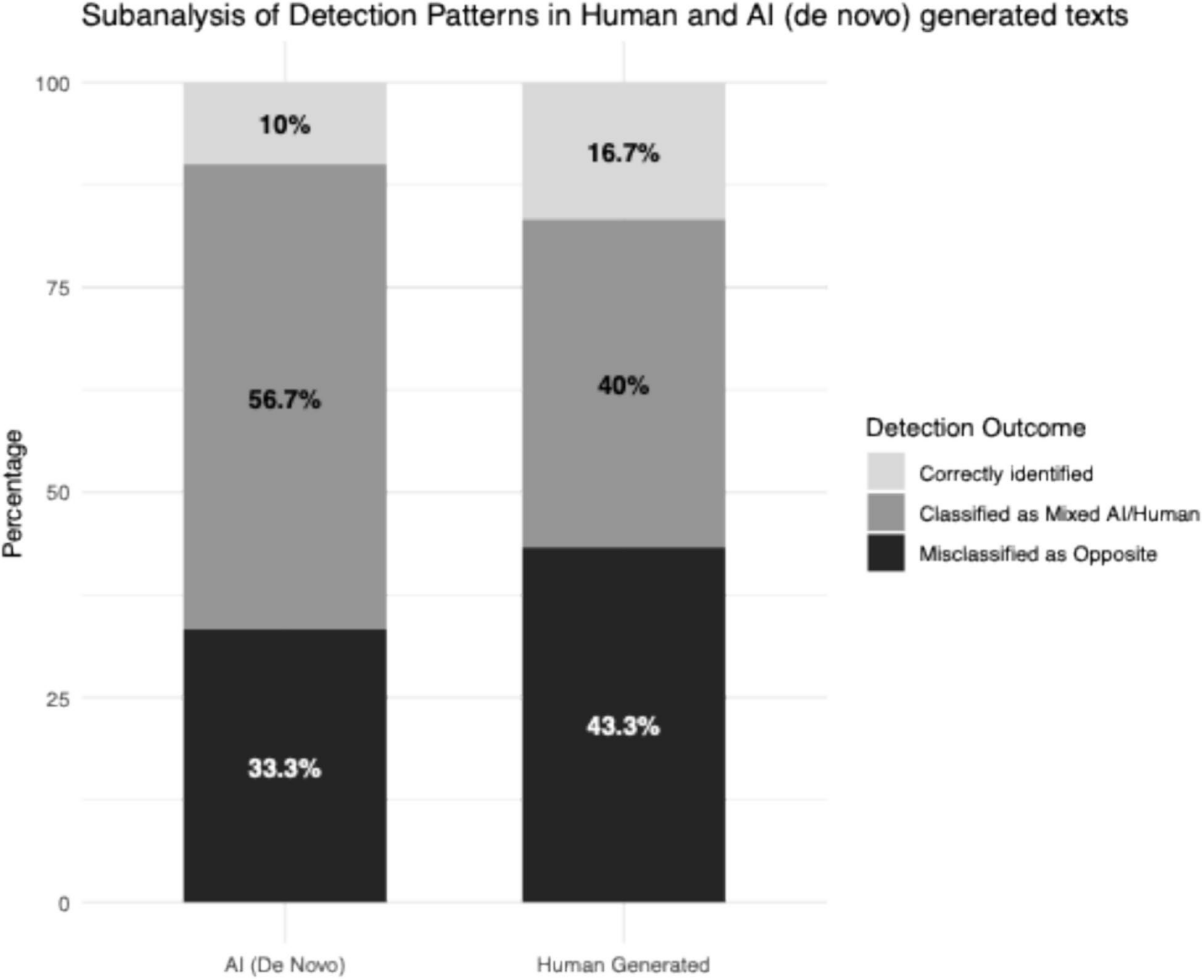
Detection patterns amongst human and AI-generated text

The overall accuracy of human rating across 5 conditions was 19%, with 17% accuracy for human written text, 23% accuracy for AI lightly edited texts, 30% accuracy for AI heavily edited text, 17% accuracy for AI generated based on bullet points, and 10% accuracy for AI generated text (de novo) (Fig. 2). Amongst text purely written by humans, 12 of 30 were rated as mixture of human and AI, and the FP rate for the remaining 18 was 13/18 (72.2%), ranging from 43.4% (best-case scenario) to 83.3% (worst-case scenario) in sensitivity analyses. Amongst text generated de novo by AI, 17 of 30 were rated as mixture of human and AI, and the FN rate for the remaining 13 was 10/13 (76.9%), ranging from 33.3% (best-case scenario) and 90.0% (worst-case scenario) in sensitivity analyses. (Fig. 3, Table 2). The difference of human rating accuracy between the 5 conditions was not statistically significant (*p* = 0.34) (e-Table 3). The agreement between human and AI tool detection was extremely poor for all three tools (ICC = 0 for all). The Blant-Altman plot indicated no systematic bias, but severe proportional bias with limit of agreement from −117 to + 119 for a scale range from 0 to 100. (e-Fig. 2, Additional File).

**Table 2:** Accuracy of human detection amongst 5 conditions

Comparison	OR (95% CI)	*p*-value
Human generated text	1.00 (Reference)	—
AI lightly edited vs Human generated text	1.52 (0.42, 5.51)	0.52
AI heavily edited vs Human generated text	2.14 (0.63, 7.30)	0.23
AI generated based on bullet points vs Human generated text	1.00 (0.26, 3.84)	> 0.99
AI generated (de novo) vs Human generated text	0.56 (0.12, 2.63)	0.452
Compared with null model	χ^2^ = 4.5, df = 4, *p* = 0.344	

## Discussion

Our study demonstrates variability in the performance of three AI detection tools across five categories of generated text, ranging from human-generated to fully AI-generated text. We also found that humans are unable to reliably differentiate human-generated from AI-generated text, with an accuracy of 10% for AI-generated text and 17% for human-generated text, which are below the level expected from an essentially random process which is 20%. Furthermore, even after removing all ambiguous cases the false positive and false negative rates remained higher than 70%, and agreement between AI detection tools and human ratings was extremely poor. These results strongly suggest alternative tools or a revision of these tools are needed to reliably distinguish AI-generated text.

Although difficult to detect by humans, significant differences do exist between AI-generated versus human-generated text. AI-generated text has lower perplexity, meaning the text tends to follow common linguistic patterns and is therefore more predictable in nature [13, 14]. AI-generated text also tends to be more uniform in sentence length and structure (i.e. low burstiness), along with more frequent use of similar phrases. Indeed, this is to be expected given that AI-generated text is stochastically produced based on average, common patterns in the training dataset. In contrast, human writing tends to have significantly higher variance, with higher perplexity (e.g. unexpected word choices), and greater variation in sentence length, structure and types of phrases [13].

AI detection tools capitalize on these differences to determine whether text is AI-generated or human-generated. AI detection tools work in a number of different ways: (a) through analysis of writing structure, style, semantic meaning, linguistic features and predictability; (b) comparison of text to existing datasets of content to recognize patterns differentiating AI-generated from human-generated content; and/or (c) identification of hidden markers embedded by generative AI tools [13, 15]. Accuracy of AI detection tools is dependent upon several factors, such as properties of the LLM used to generate the text, length of the content, and the degree of human editing [14]. Even the most accurate AI detectors can only differentiate between AI or human-generated text, as they are not designed to determine the originality or accuracy of content [8].

Since the advent of generative-AI based LLMs, researchers have explored the ability of AI detection tools to accurately identify AI-generated text [8–12]. In a systematic review covering basic sciences, medical sciences, and English language studies, Chaka et al. identified 17 articles evaluating the performance of AI detection tools and concluded there was inconsistency in the accuracy of these tools [8]. One study evaluating the accuracy of 16 publicly available AI detectors reported accuracy ranging from 63–100%, with false negative rates as high as 36% (i.e. AI text rated as human), and false positive rates between 10–14% (i.e. human text rated as AI) [11]. Weber et al. assessed 14 different AI detection tools, and found the overall accuracy ranged from 43–81%, with a bias towards classifying output as human written over detection of AI-generated text (i.e. false negatives) [10]. In this study, false negative rates ranged from 8–100%, while false positive rates ranged from 0–50%, depending on the AI detection tool used. The AI detection tools we evaluated in our study fared somewhat better. The assessed tools reliably identified higher percentages of AI-based content across a hierarchy of conditions, with excellent inter-rater reliability between the two highest-performing tools (i.e. ZeroGPT and PhraslyAI), cautiously supporting their value.

In terms of human performance, the limited data available suggests that this is close to random guessing [16–18], with a tendency towards rating text as human-generated [16]. In our data the overall accuracy was 19%, which is also consistent with random guessing (i.e. 1 in 5 chance of an accurate guess), further validating the notion that humans are unable to reliably detect the use of AI in writing. While there are differences in AI versus human-generated text, it is unsurprising that human accuracy is low given that one of the primary motivators behind the development of LLM’s was the creation of systems that could generate convincing conversation. It is worth noting, that the human raters in our study were not specifically trained to detect these differences. However, the raters in our study were chosen specifically because of their speciality knowledge in simulation, which would theoretically help them in identifying inaccuracies or hallucinations. Future research could explore efforts to train human raters and determine if this affects the accuracy of AI detection.

There are several practical implications of this study for the healthcare simulation community. First, with the exception of Grammarly, the tools assessed were able to detect rising levels of AI use across the five study conditions. In our previously published work, we noted that many of the potential ethical concerns with AI in academic writing stem from its use to generate novel ideas or text, and not when it is used to perform more basic editorial functions such as restructuring for grammar and flow [6]. The conditions utilized in our study were designed to reflect that progression, with condition 2 (light editing) representing a more ethically sound approach, condition 3 (heavy editing) inhabiting a grey area requiring a degree of discernment, and conditions 4 and 5 (producing de-novo text based on bullet points and/or a simple query) representing ethically suspect approaches that are best avoided. Given the ability of the AI detection software to discriminate between the conditions, it thus may be possible for journal editorial staff to utilize percentage of AI use to flag potentially ethically suspect generative AI uses in submitted works, prompting a discussion with the authors. While we would hesitate to establish a firm cutoff for this, our data suggests that percentages of AI use within a paper above 40% should potentially prompt a closer look by editorial staff. Further work is needed to establish the value and nuances of this potential approach.

This possibility leads to further considerations regarding how best to globally address AI use at the editorial level. While the use of AI tools to support academic writing is not entirely unethical, scholars should carefully consider how these tools are used and reported prior to submitting manuscripts for publication, and journal editors should provide clear guidance on what is considered acceptable use and what is not [6, 10]. While journal reviewers and editors should not solely rely on AI detection tools to evaluate appropriate use within a manuscript, their use should be strongly considered as part of the peer review process [9]. Care must be taken, of course, when interpreting these assessments, as false positives may lead to wrong accusations of unethical AI use and false negatives will allow scholars to evade detection of AI use in generating content for their manuscript [10]. Complementing AI detection tools with other modes of assessment will thus be needed. While human reviewers would seem like the natural solution [8], our study suggests that humans are not able to differentiate human versus AI written content. Deliberate verification of all cited articles can also help, given the propensity of AI to hallucinate academic sources [19]. Ultimately, journals will need to develop their own unique approach to AI detection, and if they use AI detection tools, thresholds for AI-generated content will need to be established and shared with the academic community.

Our study has several limitations. The results we obtained are limited only to the three open access AI detection tools used in our study. Other AI detection tools exist, including those that require subscription fees, and they may perform differently than the ones we assessed in our study. We also used very specific and short prompts when asking ChatGPT4o to generate or edit text. Other types of prompts may produce text with different proportions of AI generated text; as would the use of LLMs other than ChatGPT4o. For condition 4, where we deconstructed human written text into bullet points, there may have been variability in the manner text was converted into bullet points, and this may have affected the results. However, we believe this variability is representative of how academic writers will use ChatGPT4o in this manner. An additional limitation is that there are other ‘conditions of use’ that we did not test; for example, AI-generated text with subsequent human manual edits. We decided against including this condition as it was extremely difficult to standardize the nature and extent of human manual edits, but such actions could effectively “mask” potentially unethical uses and will need to be addressed in future studies. Finally, we elected not to ‘train’ our human raters on recognizing the patterns of AI generated text. This was done specifically to mimic a ‘typical’ academic writer, who we assume is unaware of these intricate differences between AI and human written text.

## Conclusion

When tested across five conditions of generative AI use likely to be found in future scholarship, existing open-access AI content detectors were able to distinguish escalating use of AI in the text with some variation when compared to each other. Human detection fared no better than chance. This escalating use correlates with ethical concerns regarding LLM use in academic writing identified in prior publications. Relative differences in the scores generated by these tools may thus have relevance to journal editors wishing to monitor use, but significant discretion must be used given variations in performance between tools. We encourage journal editors to develop proactive, transparent plans regarding acceptable AI use as well as processes to adjudicate conflict.

## Supplementary Information

Additional file 1. Title: eFigure 1 and eFigure 2. Description:eFigure 1 - Bland-Altman Plot for 3 AI Detection Tools and eFigure 2 - Bland-Altman Plot for 3 AI Detection Tools vs. Human Raters.

## Data Availability

Data available from authors upon request.

## Abbreviations

AI: Artificial Intelligence
ChatGPT: Chat Generative Pre-trained Transformer
LLMs: Large Language Models
ANOVA: Repeated measures analyses of variance
ICC: Intraclass correlation coefficient
FP: False positive
FN: False negative
