# Supplementary material for: Ability of AI detection tools and humans to accurately identify different forms of AI-generated written content
Source: Adv Simul (Lond). 2025 Nov 22;10:66. doi: 10.1186/s41077-025-00396-6 (PMC12752165; doi:10.1186/s41077-025-00396-6)

Additional File

eFigure 1 – Bland-Altman Plot for 3 AI Detection Tools

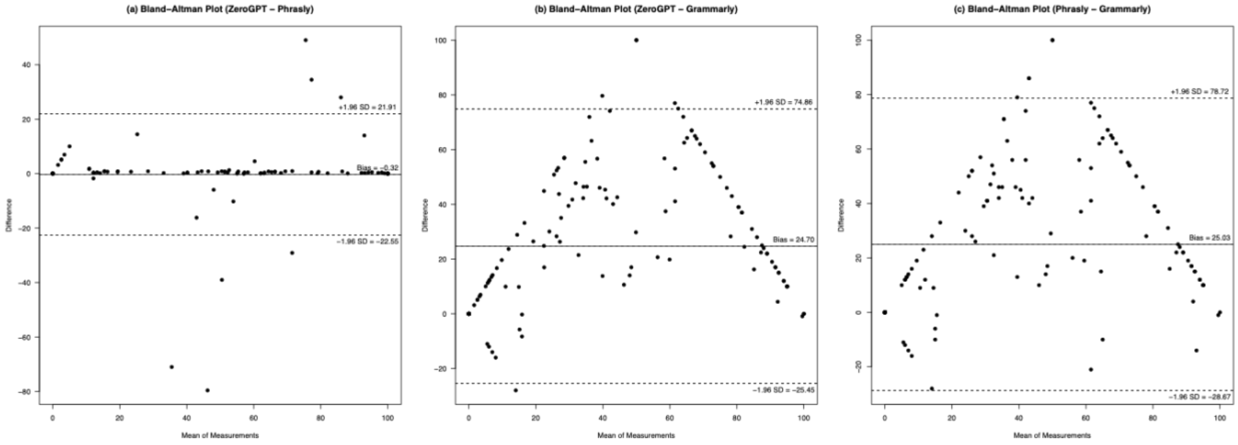

eFigure 2 - Bland-Altman Plot for 3 AI Detection Tools vs. Human Raters

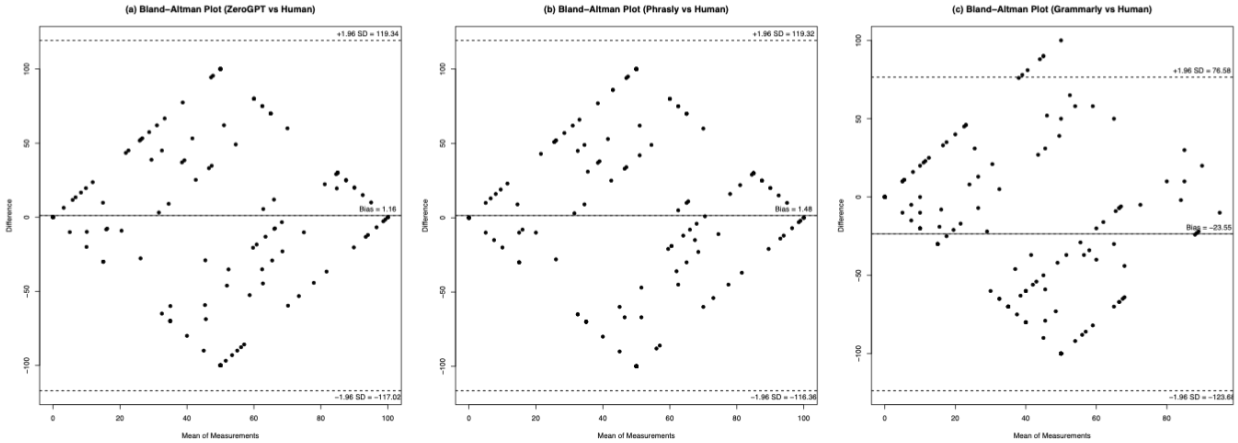

Supplement: Supplementary file 1 — Additional file 1. Title: eFigure 1 and eFigure 2. Description:eFigure 1 - Bland-Altman Plot for 3 AI Detection Tools and eFigure 2 - Bland-Altman Plot for 3 AI Detection Tools vs. Human Raters. [file 41077_2025_396_MOESM1_ESM.pdf]
